# Neural correlates of cognitive improvement over time in patients newly diagnosed with bipolar disorder

**DOI:** 10.1016/j.nsa.2026.107008

**Published:** 2026-04-29

**Authors:** Hanne Lie Kjærstad, Sebastian Vestergaard Segerlin, Julian Macoveanu, Klara Coello, Sharleny Stanislaus, Klaus Munkholm, Maria Faurholt-Jepsen, Vibeke H. Dam, Maj Vinberg, Lars Vedel Kessing, Kamilla Woznica Miskowiak

**Affiliations:** a Neurocognition and Emotion in Across Disorders of the Brain (NEAD) Centre, Psychiatric Centre Copenhagen, Mental Health Services, Capital Region of Denmark, Denmark; b Copenhagen Affective Disorder Research Centre (CADIC), Psychiatric Centre Copenhagen, Frederiksberg Hospital, Mental Health Services, Capital Region of Denmark, Denmark; c Neurobiology Research Unit, Copenhagen University Hospital, Rigshospitalet, Denmark; d Department of Clinical Medicine, Faculty of Health and Medical Sciences, University of Copenhagen, Denmark; e The Early Multimodular Prevention and Intervention Research Institution (EMPIRI), Mental Health Centre, Northern Zealand, Denmark

**Keywords:** Cognition, Bipolar disorder, Functional magnetic resonance imaging, Longitudinal

## Abstract

There is currently no known effective long-term treatment for cognitive impairments in bipolar disorder (BD) and understanding of the neurobiological mechanisms underlying cognitive impairments in BD is lacking. Identifying potential biomarkers for cognitive improvement is essential to aid targeted interventions. This study investigates the neural correlates of cognitive improvement versus lack of improvement over time in BD. Forty-six patients newly diagnosed with BD who recently began treatment in a specialized outpatient clinic and 38 healthy control individuals (HC) were assessed with a battery of neuropsychological tests and a verbal n-back working memory test during functional magnetic resonance imaging (fMRI) at baseline and follow-up (mean: 15 months). Patients were divided into two groups: those who improved cognitively over time, defined as global cognition change ≥ .2 SD, (BD+: n = 27) and those who showed a lack of normative improvement (BD-: n = 19). BD + showed hypoactivity in dorsolateral prefrontal cortex (dlPFC) and dorsomedial prefrontal cortex (dmPFC) compared to HC, both at baseline and follow-up. Hypoactivity in these prefrontal regions correlated with poorer out-of-scanner working memory and executive function for BD- and HC, but not for the BD + group. While hypoactivity did not predict change in cognition for BD+, it did predict improvement in functioning for both patient subgroups. The findings from this exploratory study suggest that dlPFC hypoactivity may identify those who are likely to respond well to treatment and show an improvement in functioning over time.

## Highlights

• Patients with BD were divided into groups based on cognitive performance over time.
• 59% experienced cognitive improvement (BD+), while 41% remained stable (BD-).
• BD + exhibited persistent working memory-related dPFC hypoactivity.
• dmPFC hypoactivity predicted subsequent improvement in functioning.

## 1 Introduction

Cognitive impairments are a core feature of bipolar disorder (BD) that persist during remission (Miskowiak et al., 2026). Approximately half of patients with BD show cognitive impairments in processing speed, attention, memory, and executive functions (Bourne et al., 2013; Van Rheenen et al., 2020). These impairments affect patients’ quality of life and daily functioning and are linked to lower academic attainment and high unemployment rates (Miskowiak et al., 2018). Yet, despite much research in the area, there are currently no known effective long-term treatments for cognitive impairments in BD (Miskowiak et al., 2019). This may be due to a lack of understanding of the neurobiological mechanisms underlying cognitive improvements in BD (Macoveanu et al., 2018).

Studies investigating the neural correlates of cognitive impairments in BD have generally found a failure to suppress the default mode network (DMN) during cognitive tasks (Alonso-Lana et al., 2016a, 2016b; Wu et al., 2014; Zarp Petersen et al., 2022) and hypoactivity in dorsolateral prefrontal cortex (dlPFC) during working memory-related tasks (Alonso-Lana et al., 2016b; Zarp Petersen et al., 2022; Macoveanu et al., 2021, 2023; McKenna et al., 2014) (for a systematic reviews, see (Saldarini et al., 2022; Pérez-Ramos et al., 2024)). However, there is a considerable degree of inconsistency between studies (Saldarini et al., 2022), with some studies having found hyperactivity in dlPFC during cognitive tasks (Costafreda et al., 2011; Glahn et al., 2010). These contradicting findings may be explained by the highly heterogeneous nature of BD with evidence supporting the existence of subgroups with differing cognitive performance (Bora et al., 2016; Burdick et al., 2014a). Studies using data-driven cluster analysis have generally identified three distinct subgroups of patients with BD: I) a cognitively normal group whose performance is comparable to healthy controls group (range 22-44%), II) an intermediate group showing mild to moderate cognitive impairment and/or selective cognitive difficulties (range 29-51%), and III) a group showing global cognitive impairments (range 23-40%) (Bora et al., 2016; Lima et al., 2019; Kjærstad et al., 2022; Burdick et al., 2014b). In line with this, a systematic review found that subgroups of BD patients with cognitive impairments showed hypoactivity in dlPFC, suggesting that dlPFC hypoactivity reflects reduced cortical capacity whereas hyperactivity reflects reduced cortical efficiency requiring patients to recruit more neuronal resources to maintain performance (Miskowiak et al., 2019).

Only a few studies have investigated the neural underpinnings of cognitive improvement over time in BD. With regards to studies of *specific* treatment-related cognitive improvement following pharmacological and psychological treatments that directly target cognition, results have consistently shown a modulation of dorsal PFC activity and attenuation of DMN hyperactivity with improved cognitive performance (Miskowiak et al., 2019, 2022). Patients treated with erythropoietin (Miskowiak et al., 2016a) or cognitive remediation therapy (Meusel et al., 2013) exhibit enhanced task-related working memory activity in the dlPFC with improved cognitive performance. Additionally, one study investigating neuronal and cognitive predictors of improved executive function found that treatment-related improvement was predicted by more working memory related dlPFC hypoactivity at baseline (Miskowiak et al., 2021). Moreover, *nonspecific* cognitive improvement following symptom reduction after mania in BD was accompanied by decrease in left amygdala activation during an inhibitory control task (Kaladjian et al., 2009).

However, the precise locations and direction of the neural activity underlying cognitive impairment – as well as cognitive improvements over time – are still not fully understood. A neurocircuitry-based biomarker for pro-cognitive effects in BD is important to enable reliable and valid prediction of treatment-related modulation, of which the dlPFC and DMN currently remain the most promising markers for pro-cognitive effects (Miskowiak et al., 2019). Cognitive subgroups may also play an important role in determining the cognitive trajectories. Whereas longitudinal studies looking at cognitive trajectories for patients with BD as one group have found no significant change over time (Torres et al., 2020; Knorr et al., 2021; Szmulewicz et al., 2020; Kjærstad et al., 2023), studies looking at cognitive trajectories for subgroups of patients with BD have found a significant improvement over time for the globally impaired patients (Kjærstad et al., 2022; Ehrlich et al., 2022). Accordingly, a neurocircuitry-based biomarker for cognitive improvement in BD can aid targeted interventions to improve cognition which is currently lacking (Miskowiak et al., 2019). Also, the identification of neural changes associated with lack of cognitive improvement, or cognitive decline over time, can be used to proactively allocate additional resources to interventions that specifically target cognition for that group.

This study is the first to investigate the functional neural correlates of cognitive improvement in cognitive subgroups in BD. We here investigated the neural changes over time associated with cognitive improvement in newly diagnosed patients with BD compared to healthy controls. We hypothesized that cognitively impaired patients would exhibit hypoactivity in regions within a cognitive control network – particularly the dlPFC - which would normalize over time for the subgroup of patients who showed cognitive improvement (Miskowiak et al., 2019).

## 2 Methods

### 2.1 Study design and participants

All patients newly diagnosed with BD were recruited at the Copenhagen Affective Disorder Clinic. The clinic receives patients from the entire Capital Region of Denmark, comprising a catchment area of 1.8 million people and provides assessment and treatment service specifically for patients with newly diagnosed/first episode BD. Patients received specialized treatment comprising evidence-based psychopharmacological treatment and group psychoeducation (Kessing et al., 2013). All patients included in the study were referred to the clinic as newly diagnosed, i.e., onset of BD for the first time and within two years and were routinely invited to participate in the Bipolar Illness Onset (BIO) study (Kessing et al., 2017). Patients were initially diagnosed with BD based on the International Classification of Diseases (ICD-10) by a psychiatrist (World Health, 1993). Patients who were deemed eligible were asked to take part in the study, where a medical doctor or psychologist verified the diagnoses with the Schedules for Clinical Assessment in Neuropsychiatry (SCAN) (Wing et al., 1990). BD type I or II was determined based on the Diagnostic and Statistical Manual of Mental Disorders (DSM-4) (APA A. American PsychiatricD.S.M.T.F. American Psychiatric Association, 2013). Inclusion criteria were age between 18 and 60 and either full or partial remission, defined as a score of ≤14 on Hamilton depression rating scale (HDRS-17) (Hamilton, 1960) and ≤14 on Young Mania Rating Scale (YMRS) (Young et al., 1978). Healthy control individuals (HC) were matched on group level in terms of age and sex and recruited through the University Hospital, Rigshospitalet, Blood Bank. HC were excluded if they had a history of mental or substance abuse disorder in their family (first-degree relatives). Exclusion criteria for all participants were contraindications for MRI (e.g., metal implants, pregnancy etc.), neurological disorders, severe brain injury, current substance abuse, or current severe somatic illness. Prior to participation, informed consent from participants was obtained.

### 2.2 Procedure

The participants completed two neuropsychological testing sessions. Both sessions included clinical evaluations of depressive and manic symptoms (HDRS-17 and YMRS), assessment of functioning, and quality of life, magnetic resonance imaging, and neurocognitive testing.

### 2.3 Measures

#### 2.3.1 Socio-demographic data

Demographic data were collected at baseline and included age, years of education, and sex. Additionally, a verbal intelligence quotient (IQ) was estimated by the Danish version of the National Adult Reading Test (DART) (Nelson et al., 1978). Lastly, the participants functioning, and quality of life were determined by Functional Assessment Short Test (FAST) (Rosa et al., 2007) and European Quality of Life 5-Domain (EQ-5D) (EuroQol, 1990)**.**

#### 2.3.2 Neurocognitive assessment

The tests used to assess cognitive functioning at baseline and follow up were: Trail Making test-A and B (TMT-A/TMT-B) (Battery, 1944), the Rey Auditory Verbal Learning Test (RAVLT) (Schmidt, 1996), the letter number-sequencing subtest from Wechsler's adult intelligence scale 3rd edition (WAIS-III) (Wechsler et al., 1997), Coding and Digit Span Forward from the Repeatable Battery for the Assessment of Neuropsychological Status (RBANS) (Randolph et al., 1998), letter verbal fluency (S and D) (Borkowski et al., 1967), the Spatial Working Memory (SWM) test and the Rapid Visual Information Processing (RVP) test from the Cambridge Neuropsychological Test Automated Battery (CANTAB). Alternate version of the RAVLT (list AB, GeAB, and Cr-AB) and RBANS coding (versions A and B) were counterbalanced at both baseline and follow-up to minimize practice effects.

### 2.4 Functional magnetic resonance imaging

#### 2.4.1 Verbal n-back working memory paradigm

The participants completed a verbal n-back task in the MRI-scanner. The task consists of four conditions (0-back, 1-back, 2-back, and 3-back) where the participants are presented with a sequence of letters on the screen. In the 0-back condition, the participants were asked to press a pad every time they saw the letter X. In the 1-back condition, they were asked to indicate when the letter was the same as the previous letter. In the 2-back condition, they were asked to indicate when the letter was the same as two trials before (e.g., **A** B **A**). In the 3-back condition, they were asked to indicate when the letter was the same as 3 trials before (e.g., **A** B Z **A**).

### 2.5 Statistical analyses

#### 2.5.1 Processing of neurocognitive data

Raw scores for each test were transformed to z-scores by using baseline means and standard deviation from the HCs. In order for the Z-scores to reflect better performance, the TMT-A and B, RVP, and SWM scores were reversed. The Z-scores were then averaged into four domains: 1) Processing speed (RBANS coding, TMT-A, digit span), 2) sustained attention (CANTAB RVP), 3) verbal learning and memory (RAVLT list IV, immediate and delayed recall, and recognition), and 4) working memory and executive functions (CANTAB SWM between errors and strategy, TMT-B, verbal fluency S and D, WAIS-III letter-number sequencing). An average for all domains was calculated to obtain an overall measure of global cognition. Participants’ change (delta) scores were calculated by subtracting the global cognition z-score at follow-up from baseline. Finally, patients were grouped into those who showed *cognitive improvement* (BD+) and those who showed *lack of normative improvement* (BD-) over the follow-up time. In line with the recommendations from the International Society of Bipolar Disorder (IBSD) Targeting Cognition Taskforce (Miskowiak et al., 2017), cognitive improvement was defined as global cognition delta change ≥.2 and lack of selective impairment (change score ≤0) in two or more cognitive subdomains, whereas lack of normative improvement was defined as delta change score of ≤0 in either global cognition or in two or more domains.

#### 2.5.2 Statistical analysis of behavioral data

ANOVA with Sidak correction for multiple comparisons, nonparametric Kruskal-Wallis test, and Pearson's chi-square was used to assess neurocognition, demographic, and clinical characteristics between the two groups. Statistically significant differences between the groups (p < .05, two tailed) were followed up with pair-wise comparisons. Differential change in functioning, cognition, and mood symptoms were analyzed with analyses of covariance (ANCOVA) with group as between-subjects factor and time as within-subjective factor, adjusting for differences in follow-up times between participants. Statistically significant 3 (group: BD+, BD-, HC) x 2 (time: baseline vs. follow-up) interactions were followed-up with pairwise interaction analyses and t-tests to investigate within group change over the follow-up time. Sidak correction for multiple comparisons was used.

### 2.6 fMRI analysis

#### 2.6.1 Pre-defined anatomical masks

A volumes of interest (VOI) mask was used for the cognitive control network (CCN) based on previous meta-analytic finding (Emch et al., 2019), which included superior and middle frontal gyrus, superior parietal lobule, postcentral gyrus, angular gyrus, precuneus, supramarginal gyrus, and parietal operculum. Regions were based on the Harvard-Oxford Cortical Structural Atlas thresholded at 30%.

#### 2.6.2 Pre-processing and first-level analysis

FMRI Expert Analysis Tool (FEAT) v6.0 from FMRIB software (FSL: https://fsl.fmrib.ox.ac.uk/fsl/fslwiki) was used for pre-processing and first-level analysis. Pre-processing included brain extraction, field distortion correction using field map image, linear and nonlinear registration to structural space, motion correction, spatial smoothing, and spatial normalization to the Montreal Neurological Institute (MNI) standard space. For each participant registrations and functional images were inspected.

First-level analysis used general linear model and included four conditions: 0-back, 1-back, 2-back, and 3-back. Four contrasts were used: 3-back vs. 0-back, 2-back vs. 0-back, 1-back vs. 0-back, and a contrast with the linear working memory (WM) load (1.5∗3-back +.5∗2-back −.5∗1-back −1.5∗0-back). If participants moved excessively (mean frame-wise displacement >.2 mm), they were excluded from the analyses (Woolrich et al., 2004).

#### 2.6.3 Group-level analysis of fMRI data

The general linear models included the four contrasts from the first-level analysis, i.e., 3-back vs. 0-back, 2-back vs. 0-back, 1-back vs. 0-back, and the linear effect of WM load, with the latter being the contrast of interest. All group-level analyses were conducted using the cognitive control network VOI as well as on a whole-brain level for explanatory purposes. The significance level for the clusters were set to p < .05 corrected for multiple comparisons based on a cluster-forming threshold of Z = 2.56 (p < .005).

First, the baseline differences between HC and the two subgroups were estimated. Then, longitudinal analyses were used to investigate (I) group differences independent of time (main effect of group); and (II) group differences in WM-related activity over time (group-by-time interaction) between either BD + vs BD-, BD + vs HC or, BD-vs HC. The first-level contrasts for each participant at both timepoints was used for the second-level analyses which examined mean response and delta change score. Lastly, the three level mixed effects analyses included the contrasts from the second level and examined the differential neural activity associated with change in WM performance. Differences between the three groups were assessed using F-test and pairwise comparisons between the groups were explored. To account for differences in time between baseline and follow-up, all models included a regressor to control for the follow-up time in years (Woolrich et al., 2004).

#### 2.6.4 Post-hoc analyses

Mean percent BOLD signal change within significant clusters was extracted using the featquery tool in FSL for visual illustration of the effects. Extracted BOLD signal change from these clusters was also used for exploratory post-hoc analyses. Pearson and Spearman's Rho correlation analyses were conducted to investigate the associations between baseline neural activity during the working memory n-back task and the behavioural domain of working memory and executive function first across the entire cohort, then within each of the three groups. For patients, we further investigated the association between extracted WM-related fMRI BOLD response from the identified clusters within the CCN and medication status. Multiple regression analyses were conducted to explore whether neural activity during working memory at baseline was associated with (i) change in global cognition or (ii) change in functioning, respectively. Extracted BOLD signal change from significant clusters in the CCN analyses were entered as predictor variables. Analyses controlled for age, sex, years of education, change in subsyndromal symptoms, and time between scans. The analysis with change in functioning (FAST) as outcome variable additionally adjusted for change in global cognition.

## 3 Results

### 3.1 Participants and cognitive profiles

This study included baseline and follow-up fMRI data from 84 individuals: 46 patients with newly diagnosed BD and 38 HC (Table 1). Two patients were excluded due to incomplete fMRI files. Mean delay of follow-up assessment was 15 (±5) months.

**Table 1:** Demographics and clinical characteristics in patients with bipolar disorder whose cognition improved over the follow-up time (BD+), patients with who showed a lack of normative improvement (BD-) over time and healthy control individuals (HC) across both baseline and follow-up.

	Baseline							Follow-up							Longitudinal	
	BD+	BD-	HC	P-value	Pairwise comparison			BD+	BD-	HC	P-value	Pairwise comparison			Main effect of group	Group × time interaction
					BD + vs. BD-	BD + vs. HC	BD- vs. HC					BD + vs. BD-	BD + vs. HC	BD- vs. HC		
**Demographic variables**																

*N*	27	19	38					27	19	38						
Sex, *n* (% female)	17 (63%)	11 (58%)	23 (61%)	.940												
Age	30 [24:35]	27 [25:35]	25 [22:35]	.596												
Years of education	15.17 (2.72)	15.42 (3.92)	15.32 (2.30)	.955				15.59 (2.58)	15.74 (3.12)	15.57 (1.92)	.968				.974	.815
Time Between baseline and follow-up, months								15.7 (4.8)	13.7 (4.9)	15.3 (4.9)	.46					
IQ	113.99 (4.21)	111.87 (5.45)	112.99 (4.89)	.364												
HDRS	4 [1:8]	5 [2:8]	0 [0; 1.3]	**<.001**	.720	**<.001**	**<.001**	4 [1:8]	2 [0:6]	0 [0:2]	**<.001**	.318	**<.001**	**.003**	**<.001**	.350
YMRS	1 [0:4]	1 [0:6]	0 [0:0]	**.001**	.963	**<.001**	**.007**	0 [0:2]	1 [0:2]	0 [0:1]	**.036**	.316	.125	**.010**	**.001**	.347
EQ-5D	.86 [.78:1]	.87 [.81:1]	1 [1:1]	**<.001**	.372	**<.001**	**.002**	1 [.86:1]	.86 [.80: .90]	1 [1:1]	**<.001**	.181	**.016**	**<.001**	**<.001**	.078
BMI	24.01 (3.23)	27.46 (4.90)	23.53 (3.14)	**<.001**	**.003**	.60	**<.001**	24.47 (2.99)	27.34 (5.26)	23.80 (3.10)	**.005**	**.01**	.47	**.001**	**.009**	.40
Smoking status, n (%) current smokers	7 (26%)	6 (32%)	7 (18%)	.27												
Alcohol, units per week	3 [0:6]	1 [0:2.5]	5 [2:8]	.89												

**Clinical characteristics**																

BD Type, *n* (% type II)	19 (70%)	14 (70%)			.806											
Illness duration^a^	4 [3:11]	6 [2:15]			.771											
Untreated illness^b^	4 [1:10]	3 [1:14]			.982											
Lithium, *n* (%)	15 (56%)	9 (47%)			.584			8 (30.8%)	6 (33.3%)			.858				
Anticonvulsants, *n* (%)	11 (41%)	9 (47%)			.655			23 (88.5%)	13 (72.2%)			.170				
Antidepressants, *n* (%)	4 (15%)	5 (26%)			.333			0 (0%)	3 (17%)			**.031**				
Antipsychotics, *n* (%)	4 (15%)	6 (32%)			.175			10 (38.5%)	4 (22.2%)			.256				
Total number of prior mood episodes	12 [8:20]	16 [6:27]			.539			15 [9.5:21]	17.5 [6.5:28.5]			.519			.683	.810
Depression	5 [4:9]	8 [5:10]			.218			6.5 [4:11]	9 [4.5:11.5]			.382			.668	.819
(Hypo)mania, mixed	6.5 [3:11]	4.5 [1:15]			.849			7.5 [3:11]	6 [2:16]			.933			.677	.424
Total duration of prior mood episodes, days	746 [228: 1288]	458 [167: 847]			.329			784 [280:1236]	486 [162:727]			.238			.169	.894
Depression	370 [96:1050]	304 [91:608]			.378			439 [181:1108]	332 [107:588]			.190			.142	.218
(Hypo)mania, mixed	91 [34:403]	91 [20:234]			.595			91 [46:332]	91 [36:285]			.784			.470	.139

Note: Values are presented as means (standard deviations) and medians [interquartile range]. a = illness duration was calculated from first (hypo)manic/mixed episode to cognitive test; b = duration of untreated psychiatric illness was calculated from first (hypo)manic/mixed episode to time of diagnosis. Abbreviations: HDRS = Hamilton Depression Rating Scale; YMRS = Young Mania Rating Scale; EQ-5D = EuroQol-5 Domain Quality of Life; BMI=Body Mass Index; BD = Bipolar Disorder.

**Fig. 1:**
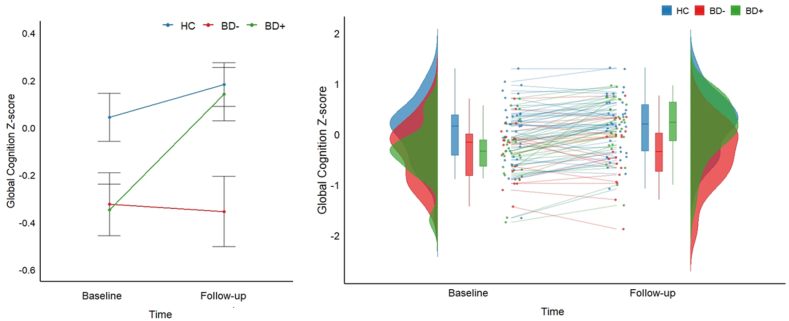
**Left:** Change in global cognition (mean z-score) over time across patients with bipolar disorder who improve over time (BD+, green), patients who show a lack of normative improvement (BD-, red), and healthy controls (HC, blue). The trajectory of cognitive change significantly differed among the groups (p < .001): HC exhibited normative improvement over time (p = .002), while BD + patients showed a significantly greater slope of improvement (within-group change over time: p < .001; group by time interaction: BD + vs. HC: p < .001). In contrast, BD-patients showed lack of normative improvement in cognition over time (within-group change p = .68; BD-vs. HC: p = .04, BD + vs. BD-: p < .001). **Right:** Distribution and individual-level cognitive course from baseline to follow-up presented with split violin plot showing dispersion, box plots and individual trajectories.

Nineteen patients with BD met the criteria for cognitive improvement (BD+) over time and 27 met the criteria for lack of normative improvement (BD-). There was a statistically significant trajectory difference between BD+, BD-, and HC in global cognition over time (F(2, 80) = 21.69, p < .001, η_p_^2^= .35): while HC showed normative improvement over time (likely due to possible retest effects) (p = .002, d = .25), BD + showed a significantly greater slope of improvement over time (within-group change over time: t = 9.32, p < .001, d = .27; group by time interaction: BD + vs. HC: F(1, 62) = 28.86, p < .001, η_p_^2^ = .31), and BD-showed lack of normative improvement in cognition over time (within-group change p = .68; BD-vs. HC: F(1, 54) = 4.62, p = .04, η_p_^2^= .08, BD + vs. BD-: F(1, 43) = 32.28, p < .001, η_p_^2^ = .43) (Table 3, Fig. 1). See supplementary material for results pertaining to each individual cognitive domain.

### 3.2 Baseline demographics, clinical characteristics, and functioning

Patients in the two BD subgroups and HC were well matched with respect to sex, age, years of education, IQ, smoking status, and alcohol use at baseline (p-values≥.27). Patients and HC significantly differed in scores of HDRS, YMRS, EQ-5D, functioning, and BMI. These group differences were driven by more subsyndromal depression and mania symptoms, lower quality of life, and poorer total functioning (FAST) in both patient subgroups compared to HC (p-values≤.007) with no statistically significant differences between the two patient subgroups (p-values≥.34). The group difference in BMI was driven by BD-patients having a higher BMI than both BD + patients and HC (p-values≤.003). There were also no statistically significant differences between the two patient subgroups in baseline clinical characteristics such as BD type, illness duration, or medication (p-values≥.09) (Table 1, Table 2).

**Table 2:** Functioning in patients with bipolar disorder whose cognition improved over the follow-up time (BD+), patients with who showed a lack of normative improvement (BD-) over time and healthy control individuals (HC) across both baseline and follow-up.

	Baseline							Follow-up							Longitudinal	
	BD+	BD-	HC	P-value	Pairwise comparison			BD+	BD-	HC	P-value	Pairwise comparison			Main effect of group	Group × time interaction
					BD + vs. BD-	BD + vs. HC	BD- vs. HC					BD + vs. BD-	BD + vs. HC	BD- vs. HC		
**Functioning**																

FAST total score	11 [7:23]	9 [4:20]	0 [0:2]	**<.001**	.454	**<.001**	**<.001**	6 [2:12]	7.5 [3.5:18.3]	.5 [0:2]	**<.001**	.376	**<.001**	**<.001**	**<.001**	**.003**
FAST autonomy	1 [0:3]	0 [0:1]	0 [0:0]	**<.001**	.345	**<.001**	**.004**	0 [0:2.23]	0 [0:2]	0 [0:0]	**.010**	.661	**.004**	**.011**	**<.001**	.681
FAST occupation	2 [0:10]	2 [0:15]	0 [0:0]	**<.001**	.689	**<.001**	**<.001**	0 [0:3.23]	.5 [0:13.5]	0 [0:0]	**.002**	.431	**.002**	**.001**	**<.001**	.024
FAST cognition	2 [1:7]	2 [1:5]	0 [0:1]	**<.001**	.335	**<.001**	**<.001**	3 [1:4.25]	2.5 [0:4]	0 [0:1]	**<.001**	.411	**<.001**	**<.001**	**<.001**	.172
FAST financial	0 [0:1]	0 [0:2]	0 [0:0]	**<.001**	.444	**<.001**	**<.001**	0 [0:1]	0 [0:1.3]	0 [0:0]	**.029**	1	**.014**	**.016**	**<.001**	.216
FAST relationships	2 [0:3]	1 [0:2]	0 [0:0]	**<.001**	.513	**<.001**	**.005**	0 [0:1]	1 [0:1]	0 [0:.3]	**.043**	.496	.082	**.016**	**.002**	**.006**
FAST leisure	0 [0:1]	0 [0:1]	0 [0:0]	**.014**	.437	**.003**	**.083**	0 [0:0]	0 [0:2]	0 [0:0]	**.017**	.106	.303	**.004**	**.016**	.109

Note: Values are presented as medians [interquartile range]. Abbreviation: FAST=Functional Assessment Short Test.

**Table 3:** Neurocognitive functioning in patients with bipolar disorder whose cognition improved over the follow-up time (BD+), patients with who showed a lack of normative improvement (BD-) over time and healthy control individuals (HC) across both baseline and follow-up.

	Baseline							Follow-up							Longitudinal	
	BD+	BD-	HC	P-value	Pairwise comparison			BD+	BD-	HC	P-value	Pairwise comparison			Main effect of group	Group × time interaction
					BD + vs. BD-	BD + vs. HC	BD- vs. HC					BD + vs. BD-	BD + vs. HC	BD- vs. HC		
**Non-emotional cognition, z-score (SD)**																

Global cognition	−.35 (.57)	−.32 (.58)	.04 (.62)	**.017**	.999	**.032**	.089	.14 (.58)	−.35 (.65)	.18 (.57)	**.005**	**.019**	.990	**.005**	**.027**	**<.001**
Processing speed	−.40 (.60)	−.41 (.65)	−.01 (.85)	.054				.06 (.60)	−.41 (.57)	.02 (.76)	**.041**	.065	.997	.067	**.071**	**<.001**
Attention	−.36 (.83)	−.38 (.95)	.02 (.95)	.157				.33 (.89)	−.06 (1.12)	.53 (.65)	.051				.082	.253
Verbal learning	−.48 (1.04)	−.21 (.93)	.12 (.85)	**.041**	.710	**.037**	.507	.09 (.96)	−.43 (1.16)	.16 (.81)	.079				.134	**.002**
Working memory and executive function	−.16 (.63)	−.29 (.69)	.04 (.63)	.162				.098 (.55)	−.51 (.75)	.01 (.60)	**.003**	**.004**	.921	**.011**	.053	**<.001**

Note: Values are presented as means (standard deviations).

### 3.3 Demographics, clinical characteristics, and functioning over time

**Fig. 2:**
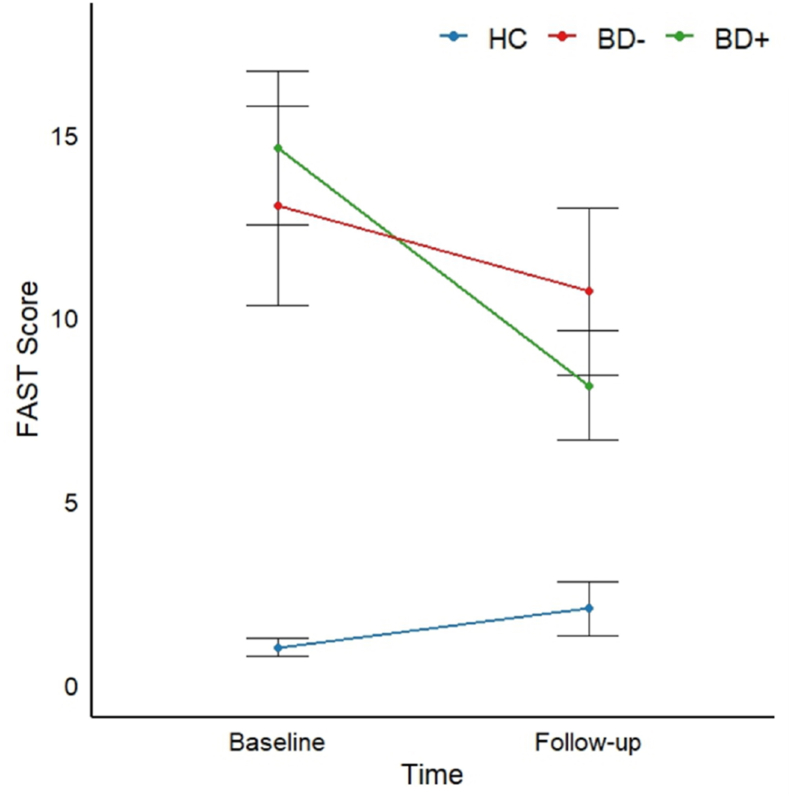
Functioning (i.e., total FAST scores) over time across patients who cognitively improve over time (BD+), patients who show a lack of normative improvement over time (BD-), and healthy controls (HC). There was a statistically significant group-by-time interaction (p = .003), which was driven by BD + patients displaying significantly greater improvement in functioning over the follow-up time (within-group change over time: p = .004) compared to HC who remained stable over time (within-group change over time: p = .16; BD + vs. HC: p < .001).

Longitudinal analyses revealed statistically significant main effects of group for HDRS, YMRS, EQ-5D, and BMI (p-values≤.009), driven by both patient subgroups generally presenting with more subsyndromal symptoms and lower quality of life compared to HC (p-values≤.02) across both time-points, with no significant differences between the two patient groups (p-values≥.96). For BMI, the statistically significant main effect of group was driven by higher BMI in BD-patients than BD + patients and HC (p-values≤.02) across both timepoints. There was a statistically significant group-by-time interaction for total functioning (F(2, 78) = 6.38, p = .003, η_p_^2^= .14), which was driven by BD + patients displaying significantly greater improvement in functioning over the follow-up time (t = 3.19, p = .004) compared to HC who remained stable over time (p = .16; BD + vs. HC: F(1, 61) = 15.37, P < .001, η_p_^2^ = .20) (Fig. 2). There was no significant trajectory difference in clinical characteristics between the two patient groups (p-values≥.14) (Table 1, Table 2).

### 3.4 fMRI analysis

#### 3.4.1 Neural activity during working memory over time

**Fig. 3:**
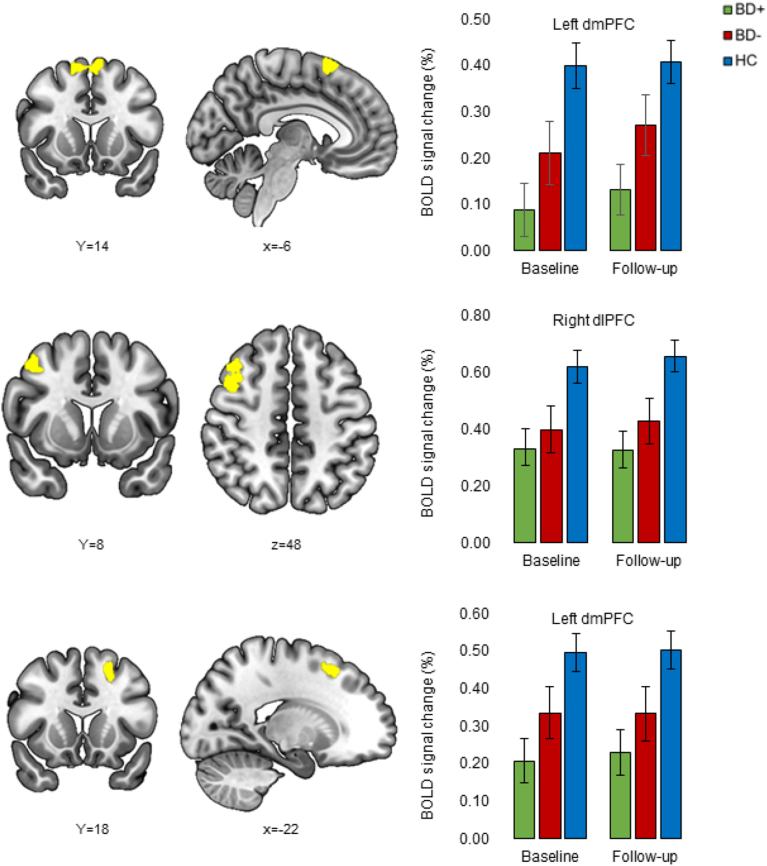
Stable decreased activity over time in two clusters in the left dorsomedial prefrontal cortex (dmPFC) and a cluster in the right dorsolateral prefrontal cortex (dlPFC) during working memory in patients with bipolar disorder whose cognition improved over the follow-up time (BD+) compared to healthy controls (HC) (p-values ≤.02), with no significant difference between patients who remained stable over the follow-up time (BD-) and HC.

CCN analysis: No significant effect of group or group-by-time interaction was observed over time. Exploratory pairwise comparisons revealed that BD + displayed hypoactivity in three clusters in the bilateral superior frontal gyrus (BA6) in the dmPFC and a cluster in the right middle frontal gyrus in the dlPFC compared to HC (p-values≤.02) across both timepoints (Table 4, Fig. 3).

**Table 4:** Main effect of group on brain activation during working memory in newly diagnosed patients with bipolar disorder whose cognition improved over the follow-up time (BD+), newly diagnosed patients with bipolar disorder who showed lack of normative improvement over item (BD-) and healthy controls (HC) across both timepoints.

Search area	Region	BA	MNI			Voxels	Peak p-value
			x	y	z		
CCN ROI							

*Main effect of group HC > BD+*							
	Left DMPFC	6	−6	14	64	508	<.000
	Right DLPFC	8	44	8	48	334	.003
	Left DMPFC	6	−22	18	50	229	.020

Whole-brain							

*F*							
	Right VLPFC	47	44	44	−14	426	.004
	Right thalamus		12	−4	4	339	.016
*Main effect of group HC > BD+*							
	Cerebellum		4	−54	−6	1476	<.001
	Left DMPFC	6	−6	14	64	644	<.001
	Right DLPFC	46	54	52	0	555	.001
	Right DLPFC	8	44	8	48	432	.004
	Left dorsal ACC	32	−6	42	12	404	.006
	Left angular gyrus	39	−42	−60	54	322	.020
	Left DMPFC	6	−22	18	50	297	.031
*Main effect of group BD+ > HC*							
	Postcentral gyrus	6	66	−6	40	443	.003
*Main effect of group BD- > BD+*							
	Right VLPFC	47	44	44	−14	516	.001

Abbreviations: BA=Broadmann Area, MNI=Montreal Neurological Institute, CCN=Cognitive control network, ROI=Region of Interest, DMPFC = dorsomedial prefrontal cortex, DLPFC = dorsolateral prefrontal cortex, VLPFC = ventrolateral prefrontal cortex, ACC = Anterior cingulate cortex.

Whole-brain analyses: The whole-brain analysis revealed a statistically significant effect of group in a cluster in the right middle frontal gyrus in the vlPFC and the right thalamus (p-values≤.02). Exploratory pair-wise group comparisons at baseline further revealed that BD + displayed hypoactivity in seven clusters compared to HC, encompassing the cerebellum, two clusters in the left superior frontal gyrus (BA6) in the dmPFC, two clusters in the right middle frontal gyrus (BA46/8) in the dlPFC, as well as the left dorsal ACC (BA32), and the left angular gyrus (BA39) (p-values≤.03). Moreover, BD + showed hypoactivity in a cluster in the right middle frontal gyrus (BA47) in the vlPFC compared to BD- (p = .001). Lastly, the analysis revealed that BD + showed hyperactivity in the postcentral gyrus compared to HC (p = .003). There was no significant group-by-time interaction effect (Table 4).

See supplementary material for baseline group differences in neural activity during working memory as well as analyses assessing entire BD cohort compared to controls.

#### 3.4.2 Correlation analyses

Across all participants, poorer baseline working memory and executive function correlated with lower signal change in the three CCN clusters (left dmPFC: r = .28, p = .009; right dlPFC: r = .34, p = .002; left dmPFC: r = .25, p = .02). However, when analysing each group separately, poorer baseline working memory and executive function correlated with lower signal change in the left dmPFC and right dlPFC for HC (left dmPFC: r = .36, p = .03; right dlPFC: r = .38, p = .02) as well as in the two left dmPFC clusters for BD- (r = −63, p = .004 and r = .50, p = .03), whereas poorer working memory and executive function was not significantly associated lower signal change with in BD + patients (p-values>.40).

With regards to medication, lower baseline BOLD response in the three CNN clusters did not significantly correlate with medication status (antidepressants, antipsychotics, anticonvulsants, lithium) in neither BD + nor BD-patients (p-values ≥.07.)

#### 3.4.3 Regression analyses

We found no evidence that baseline signal change in the right dlPFC and left dmPFC during WM was associated with change in global cognition (p-values≥.57). However, regression analyses showed statistically significant effects of right dlPFC and left dmPFC hypoactivity during WM at baseline on change in functioning (right dlPFC: F(8, 73) = 2.25, p = .03; left dmPFC: F(8, 73) = 2.30, p = .03): baseline right dlPFC and left dmPFC hypoactivity was associated with subsequent improvement in functioning (right dlPFC: β = 4.70, 95% CI [−.01; 9.42], p = .051; left dmPFC: β = 5.62, 95% CI [.23; 11.02], p = .04), although this was only at trend-level for the right dlPFC.

## 4 Discussion

This study investigated the neural correlates of cognitive improvement in patients with newly diagnosed BD over a 15-month follow-up time. Both patient subgroups presented with global cognitive impairments at baseline, but 59% (n = 27) showed cognitive improvement (BD+) and 41% (n = 19) showed a lack of normative improvement (BD-) over time, compared to HC who showed normative improvement. Furthermore, BD + patients also improved significantly in terms of functioning over the follow-up time. Surprisingly, at the neural level, BD+, but not BD-, showed hypoactivity in the dlPFC and dmPFC compared to HC, which persisted over time independent of cognitive improvement. More hypoactivity in the dlPFC and dmPFC was associated with poorer working memory and executive functioning in both BD- and HC, while this association was lacking in the BD + subgroup. Finally, more hypoactivity in the left dmPFC at baseline predicted subsequent improvement in functioning for both patient subgroups.

Our findings of persistent dorsal PFC hypoactivity in the BD + group over time were surprising and in contrast to our hypothesis. While we expected to observe impairment-related dorsal PFC hypoactivity at baseline based on reports from previous studies (Miskowiak et al., 2019), we had hypothesized a normalization of the aberrant activity over the course of treatment. One reason for this persistent hypoactivity could be that the treatments were not specifically tarting targeted cognitive function. Meanwhile, cognitive intervention studies investigating the effects of erythropoietin and cognitive remediation have found that treatment-related cognitive improvement was associated with increase in dlPFC activity (Meusel et al., 2013; Miskowiak et al., 2016b, 2021). The present study also looked at newly diagnosed patients, and it is possible that patients who have further progressed in their illness show more impairment-related dPFC hypoactivity. Alternatively, the results may reflect improvement in overall functioning rather than cognitive change. Hypoactivity in dmPFC was associated with subsequent improvement in functioning; hence, the group may better be characterized as the group that responds well to specialized treatment. The patient cohort followed specialized treatment at the clinic involving optimized pharmacological treatment and group-based psychoeducation for patients newly diagnosed with BD. It could be that BD + responded well to treatment, which led to improvement in functioning and consequently improvement in cognition.

Dorsal PFC hypoactivity may reflect a marker of treatment response and as such could identify those who are likely to respond well to treatment and improve in general functioning which, in turn, might relate to improvement in cognition. Those who do not improve may require additional treatment that specifically targets cognitive impairments. It could also be that the BD + group utilize their neuronal resources in a different way than what was expected based on existing literature. The baseline whole-brain analysis revealed that the BD + group showed hyperactivity in a cluster located in the postcentral gyrus. BD + patients may thus recruit alternative regions, such as the postcentral gyrus, during working memory tasks. Indeed, the postcentral gyrus is implicated in spatial working memory (Zhou et al., 2023), potentially suggesting BD + allocate greater resources to motor areas during completion of the WM task, which although speculative, may be associated with their improvement over time. Notably, BD patients - *at a group-level* - show the same slope of normative improvement in cognition as HC (i.e., no significant time × group interaction p = .39), highlighting the importance of investigating subgroups of patients with differing cognitive trajectories over time.

This study has several strengths. It is a longitudinal fMRI study which means that the study can uncover differences over time. Furthermore, a well-established fMRI paradigm was used as well as an extensive cognitive battery. The patients were also in full or partial remission which is an important point since mood episodes have been linked to changes in neural activity and can therefore potentially confound the results. Lastly, the patients were newly diagnosed which means that it is possible to assess the early abnormalities of the disorder. However, there are also several limitations. The relatively small sample size in this study, combined with the division into subgroups, may reduce power, limiting the generalizability of the findings. Future studies with larger samples are needed to validate these preliminary observations and enhance reproducibility. Additionally, despite being newly diagnosed, the patients had a relatively long illness duration at baseline (Median, BD+: 4 years and BD-: 6 years). Indeed, the well-known diagnostic delay in BD, typically spanning 5-10 years (Dagani et al., 2017), results in prolonged duration of untreated illness. This diagnostic delay poses challenges in achieving a homogeneous sample of “newly diagnosed” patients as some patients may have suffered from the illness during several years and introduces potentially confounding neuroprogressive effects. Moreover, most patients received psychotropic treatment (83% at baseline), which prevented comparisons between medicated and unmedicated patients in WM-related neural activity. Whereas there were overall no statistically significant differences between the two patient subgroups in medication status at baseline nor follow-up, more BD-patients received antidepressants at follow-up compared to BD+. Sensitivity analyses excluding patients receiving antidepressants at follow-up did not alter results of analyses investigating cognitive changes over time nor WM-related neural activity in the significant clusters (see supplementary material). It was a limitation that the small sample size hindered more in-depth investigations into the associations of the different medication classes with cognitive performance over time. Also, we cannot account for the cumulative effects of medication, which may affect cognitive performance both at baseline and longitudinally over the follow-up time. Thus, we cannot exclude the potential adverse effects of illness and the different treatments may further confound. The follow-up period was relatively short (mean 15 months), and it could be the reason why the study did not find any significant changes following cognitive improvement. Moreover, we cannot exclude the possibility of sampling bias due to be able to participate in two MRI scans over 18 months. In-scanner behavioural data during the N-back WM paradigm was partially corrupted in a large part of the samples due to technical issues which prevented statistical assessment. However, we were able to verify that the participants were consistently engaged throughout the task. Analyses examining associations between neural activity and working memory performance was therefore conducted using working memory and executive function tasks administered outside the scanner. Lastly, the observed improvement in global cognition among HC was likely due to practice effects, as participants became more familiar with the cognitive tasks at follow-up. However, a few cognitive tasks had different versions to minimize this effect.

## 5 Conclusion

This exploratory study investigated the neural changes of cognitive improvement and lack of normative improvement in patients with BD compared to HC over 15 months. Contrary to our hypothesis, patients showing cognitive improvement over time showed hypoactivity in dlPFC and dmPFC, which persisted across both timepoints. dlPFC and dmPFC hypoactivity correlated with poorer working memory and executive function for HC and BD-patients, while this association was lacking in the BD + subgroup. While hypoactivity in dlPFC was not associated with change in cognition, it was associated with improvement in functioning. dlPFC hypoactivity may reflect a marker of treatment response and could potentially identify those who are likely to improve functioning in response to specialized treatment for bipolar disorder. However, results are preliminary and future studies with larger samples are needed to validate the findings.

## Financial Support

The study is funded by grants from the Mental Health Services, Capital Region of Denmark, The Danish Council for Independent Research, Medical Sciences (DFF-4183-00570), Markedsmodningsfonden (the Market Development Fund, 2015-310), Gangstedfonden (A29594), Helsefonden (16-B-0063), Innovation Fund Denmark (the Innovation Fund, Denmark, 5164-00001B), Copenhagen Center for Health Technology (CACHET), EU H2020 ITN (EU project 722561), Augustinusfonden (16-0083).

## Conflict of interest

HLK has received consultancy fees from Lundbeck. MV has received consultancy fees from Lundbeck, and Janssen Cilag in the past three years. LVK has received consultancy fees from Lundbeck and Teva in the past three years. VHD has received honoraria as speaker Lundbeck in the past three years. MFJ has received consultancy fees from Janssen Cilag within the past three years. KWM has received honoraria from Lundbeck, Angelini, Gedeon Richter, and Janssen-Cilag in the past three years. The remaining authors declare no conflicts of interest.
