# Supplementary material for: Neural correlates of cognitive improvement over time in patients newly diagnosed with bipolar disorder
Source: Neurosci Appl. 2026 Apr 29;5:107008. doi: 10.1016/j.nsa.2026.107008 (PMC13158769; doi:10.1016/j.nsa.2026.107008)
Supplement: Multimedia component 1 [file mmc1.docx]

**Supplementary material**

**Results**

**Cognitive profiles**

With regards to the individual cognitive domains, there was a significant group-by-time interaction for processing speed (F(2, 80)=8.02, p<.001, η_p_^2^=.17): whereas BD+ improved over time (t=5.46, p<.001, *d*=.43; BD+ vs BD-: F(1, 43)=9.06, p=.004, η_p_^2^=.17; F(1, 62)=15.18, p<.001, η_p_^2^=.20), BD- and HC showed a lack of change over time (p-values≥.66). An interaction effect was also found for verbal learning (F(2, 80)=6.67, p=.002, η_p_^2^=.14), driven by BD+ showing improvement (t=4.86, p<.001, *d*=.60; BD+ vs BD-: F(1, 43)=9.51, p=.004, η_p_^2^=.18; BD+ vs. HC: F(1, 62)=10.35, p=.002, η_p_^2^=.14) compared to BD- and HC who showed a lack of change over time (p-values≥.23). There was also a group-by-time interaction for working memory and executive function (F(2, 80)=11.16, p<.001, η_p_^2^=.22): whereas BD- deteriorated over time compared to BD+ (t=4.13, p<.001, *d*=.24; BD+ vs BD-: F(1, 43)=24.00, p<.001, η_p_^2^=.36), BD+ improved over time compared to BD- and HC (t=3.74, p<.001, *d*=.35; BD+ vs HC: F(1, 62)=9.50, p=.003, η_p_^2^=.13). There were no significant effects of group in the ‘attention’ domain (p-values≥.08).

**Baseline differences in neural activity during working memory across BD+, BD-, and HC**

CCN analysis: At baseline, BD+ showed hypo-activation in the left superior frontal gyrus (BA6) in the dmPFC and the right middle frontal gyrus (BA9) in the dlPFC compared to HC (p-values≤.02). There were no significant differences between BD+ and BD- nor BD- and HC.

Whole-brain analysis: revealed two significant clusters in the right vlPFC and the cerebellum (p-values≤.02). Further pair-wise comparisons showed that BD+ displayed significant hypoactivity in four clusters in the left superior frontal gyrus (BA6) and right medial frontal gyrus (BA8) in the bilateral dmPFC and bilateral middle frontal gyrus (BA47/8) in the vlPFC compared to HC (p-values≤.045). Further, BD+ showed hyperactivity in the precentral gyrus (BA6) compared to HC (p=.01), whereas BD- showed hypoactivity in the cerebellum compared to HC (p<.001).

**Sensitivity analysis excluding patients receiving antidepressants at follow-up**

To examine the possible impact of the group difference in antidepressant use at follow-up, sensitivity analyses excluding patients receiving antidepressants at follow-up were conducted employing the same statistical methods described in the main analysis. At the behavioral level, analyses of global cognitive performance revealed that the significant group-by-time interaction effects remained significant (F(2, 77)=19.05, p<.001, η_p_^2^=.33), with BD- still showing a lack of normative improvement in global cognition over time (within-group change p=.94; BD+ vs. BD-: F(1, 40)=24.71, p<.001, η_p_^2^=.38). At the neural level, the three level mixed effects analyses examining differential WM-related neural activity within the CCN was repeated in FSL while excluding patients taking antidepressants at follow-up. Results prevailed with no significant group-by-time interaction effects and exploratory pairwise analyses still revealing significant hypoactivity in the three clusters in the bilateral superior frontal gyrus (BA6) in the dmPFC and a cluster in the right middle frontal gyrus in the dlPFC in BD+ compared to HC (p-values≤.02) across both timepoints. Further sensitivity analyses excluding participants receiving other medication classes (lithium, antipsychotics, anticonvulsants) were hampered by the limited sample size.

**Assessing differential change in cognitive performance over time between the entire cohort of patients with bipolar disorder and healthy controls**

*Functional magnetic resonance results*

CCN analysis: We identified no significant trajectory difference (group-by-time interaction effect) during working memory between patients with BD and HC. Three-way analyses revealed a significant group difference (i.e., main effect of group): patients displayed working memory-related hypoactivity in two clusters in the bilateral middle frontal gyri (BA6/8) in the dlPFC across both timepoints compared to HC (Table S1).

Whole-brain analyses: We found no significant group-by-time interaction during working memory. The whole-brain three-way analysis revealed significant main effects of group during working memory in three clusters, driven by BD patients displaying working memory-related hypoactivity in the right middle frontal gyrus (BA8) in the dlPFC, left superior frontal gyrus (BA6) in the dmPFC, and the cerebellum across both timepoints compared to HC. Additionally, patients with BD displayed hyperactivity in a cluster in the right precentral gyrus (BA6) across both timepoints compared to HC (Table S1).

*Neurocognition*

Analyses of neurocognitive performance across the entire BD cohort compared to controls revealed no significant differential change in cognitive performance over time between groups (group-by-time interaction, p-values ≥ .06), with the exception of processing speed (F(1,81)=4.99, p=.03) for which patients with BD significantly improved over time (p=.001) whereas HC remained stable (p=.66). Moreover, significant group differences (i.e., main effects of group) were found for global cognition (F(1,81)=5.88, p=.02), as well as the domains of attention (F(1,81)=4.44, p=.04) and verbal learning (F(1,81)=412, p=.046) – all of which were driven by patients with BD generally underperforming HC across both timepoints.

**Table S1. Main effect of group on brain activation during working memory in the entire cohort of patients with bipolar disorder compared to healthy controls across both timepoints.**

| Search area | Region | BA | MNI | | | Voxels | Peak p-value |
| --- | --- | --- | --- | --- | --- | --- | --- |
|  |  |  | x | y | z |  |  |
| CCN ROI |  |  |  |  |  |  |  |
| *Main effect of group HC > BD* | |  |  |  |  |  |  |
|  | Right DLPFC | 8 | 32 | 22 | 56 | 608 | <.001 |
|  | Left DLPFC | 6 | -40 | 6 | 56 | 188 | 0.049 |
| Whole-brain |  |  |  |  |  |  |  |
| *Main effect of group HC > BD* | |  |  |  |  |  |  |
|  | Right DLPFC | 8 | 32 | 22 | 56 | 901 | <.001 |
|  | Cerebellum |  | 6 | -58 | -10 | 483 | 0.003 |
|  | Left DMPFC | 6 | -22 | 18 | 50 | 477 | 0.003 |
| *Main effect of group BD > HC* | |  |  |  |  |  |  |
|  | Precentral gyrus | 6 | 48 | 0 | 62 | 373 | 0.01 |
